# The correlation between the number of vaginal examinations during active labor and febrile morbidity, a retrospective cohort study

**DOI:** 10.1186/s12884-020-02925-9

**Published:** 2020-04-25

**Authors:** Ohad Gluck, Yossi Mizrachi, Hadas Ganer Herman, Jacob Bar, Michal Kovo, Eran Weiner

**Affiliations:** Departments of Obstetrics and Gynecology, The Edith Wolfson Medical Center, Holon, and Sackler School of Medicine, Tel Aviv University, P.O. Box 5, 58100 Holon, Israel

**Keywords:** Vaginal examination, Febrile morbidity, Intrapartum fever, Postpartum fever, Vaginal delivery

## Abstract

**Background:**

The association between the number of vaginal examinations (VEs) performed during labor and the risk of infection is unclear. The literature regarding this issue is not consensual, and the available studies are relatively small. Therefore, we aimed to study the association between the number of VEs during labor, and maternal febrile morbidity, in a very large cohort.

**Methods:**

This is a retrospective cohort study. All women who delivered vaginally ≥37 weeks, at our institute, between 2008 and 2017 were included. Patients who underwent cesarean delivery or who were treated with prophylactic antibiotics, or had a fever ≥38.0 °C prior to the first VE were excluded. Cases of intrauterine fetal death, known malformations, or missing data were excluded as well. The cohort was divided according to the number of VEs performed: up to 4 VEs (*n* = 9716), 5–6 VEs (*n* = 4624), 7–8 VEs (*n* = 2999), and 9 or more VEs (*n* = 4844). The rates of intrapartum febrile morbidity (intrapartum fever and chorioamnionitis), postpartum febrile morbidity (postpartum fever and endometritis), and peripartum febrile morbidity (any of the mentioned complications) were compared.

**Results:**

Overall, 22,183 women were included in the study. On multivariate analysis, we found that performing 5 VEs or more during labor was independently associated with intrapartum febrile morbidity (5–6 VEs: aOR = 1.83, 95% CI (1.29–2.61), 7–8 VEs: aOR = 2.65 95% CI (1.87–3.76), 9 or more VEs aOR = 3.47 95% CI (2.44–4.92)), postpartum febrile morbidity (5–6 VEs: aOR = 1.29, 95% CI (1.09–1.86), 7–8 VEs: aOR = 1.94 95% CI (1.33–2.83), 9 or more VEs aOR = 1.91 95% CI (1.28–2.82)), and peripartum morbidity (5–6 VEs: aOR = 1.48, 95% CI (1.15–1.91), 7–8 VEs: aOR = 2.15 95% CI (1.66–2.78), 9 or more VEs: aOR = 2.57 95% CI (1.97–3.34)).

**Conclusion:**

The number of VEs performed during labor is directly correlated with febrile morbidity. Performing five or more VEs during labor is independently associated with febrile morbidity; For intrapartum and peripartum febrile morbidity the risk rises as more VEs are performed.

## Background

Vaginal examinations (VEs) are a routine part of labor progression assessment [1]. They entail the subjective impression of cervical dilatation, effacement, consistency, and presenting part position [2]. Although frequently overlooked, VEs are associated with maternal discomfort, and may negatively influence labor progress, by causing anxiety and diverting the focus of the laboring woman [2, 3]. Additionally, VEs following rupture of membranes have been demonstrated to increase the risk of chorioamnionitis [4–7]. Obstetricians are, therefore, routinely expected to weigh the need for VEs for the assessment of labor progression, against the risk of maternal discomfort and of infection with an increased number of examinations [8, 9].

Despite the World Health Organization’s recommendation for a VE every 4 hours during labor [10], a substantial number of women undergo VEs more frequently than recommended [11]. In addition, there is currently no direct evidence regarding the optimal frequency or number of VEs to minimize infectious morbidity in the mother and newborn, and minimize maternal discomfort [10]. One retrospective study found that the risk for maternal fever is not significantly increased by the number of VEs [12]. However, it was limited by a relatively small sample size, and short postpartum follow-up (up to 6 hours postpartum).

Therefore, our aim was to study the association between the number of VEs performed during labor and maternal febrile morbidities, in a very large cohort of laboring women from a single tertiary center.

## Methods

### Data collection

This was a retrospective cohort study. All women who delivered vaginally at a gestational age of 37 weeks or greater, in a single university affiliated hospital, between January 2008 and December 2017 were included. Cesarean deliveries, preterm deliveries (< 37 weeks), known fetal malformations, intrauterine fetal death, and cases of missing data were excluded. We also excluded patients who were treated with prophylactic antibiotics during labor because of known Group B streptococcus carrier status, and/ or prolonged rupture of membranes (> 18 h), and patients who had fever ≥38.0 °C prior to the first VE.

For each delivery, the number of VEs performed during labor, as well as the duration of labor (from admission to the delivery ward until the time of delivery) and the time from rupture of membranes until delivery were extracted from the computerized database. Data regarding the number of VEs performed prior to admission to the delivery ward was not extracted.

All women were admitted to the delivery ward in active labor, which was defined as a cervical dilatation ≥4 cm in the presence of regular uterine contractions, with or without rupture of the membranes. According to our departmental protocol, early amniotomy is performed in every labor (if membranes are intact), as part of an active management of labor.

The primary exposure was the number of VEs during labor as documented in the electronic medical record of each delivery. Our institutional protocol mandates full documentation of each examination. VEs were performed using sterile gloves and are primarily performed by obstetricians, residents, and midwives. A water based lubricant (Sion Biotext medical LTD. Hagoshrim, Israel) is used. Amniotomy is carried out by an obstetrician, with the hook handed to her/ him by the midwife, so the hand is not removed throughout the examination and amniotomy. During labor, vital signs (including body temperature- measured orally) were measured every 2 hours, or in case of a clinical suspicion of maternal fever (maternal or fetal tachycardia, flushing, etc.). Patients are routinely discharged from the maternity ward 2 days following vaginal deliveries.

Data regarding background and obstetric characteristics, mode of delivery, delivery complications, and postpartum complications (until the patients were discharged from the hospital) were reviewed as well. The outcomes evaluated were the following delivery complications: intrapartum febrile morbidity (intrapartum fever ≥38.0 °C and/or culture-proven chorioamnionitis), postpartum febrile morbidity (postpartum fever ≥38.0 °C from the 2nd to the 10th day following delivery, and/or culture-proven endometritis), and peripartum febrile morbidity (defined as intrapartum or postpartum). Postpartum febrile morbidities were analyzed after the exclusion of patients with intrapartum febrile morbidities.

### Statistics

Data were analyzed using SPSS statistical analysis software v23.0 (IBM Inc., USA). Continuous variables were compared by the ANOVA test, and categorical variables were compared by chi-square test. All tests were two sided, and a *p*-value < 0.05 was considered statistically significant.

In order to study the association between the number of VEs and delivery complications, the cohort was divided according to the number of VEs performed during labor as follows: up to 4 VEs, 5–6 VEs, 7–8 VEs, and 9 or more VEs.

In order to identify independent variables associated with febrile morbidity complications, we performed a multivariate logistic regression analysis. Each complication (as defined above) served as the dependent variable and the following variables served as independent variables: maternal age, diabetes mellitus, maternal body mass index (BMI), gestational age, type of delivery (normal vaginal or instrumental), duration of labor and the time form rupture of membranes to delivery.

## Results

**Fig. 1:**
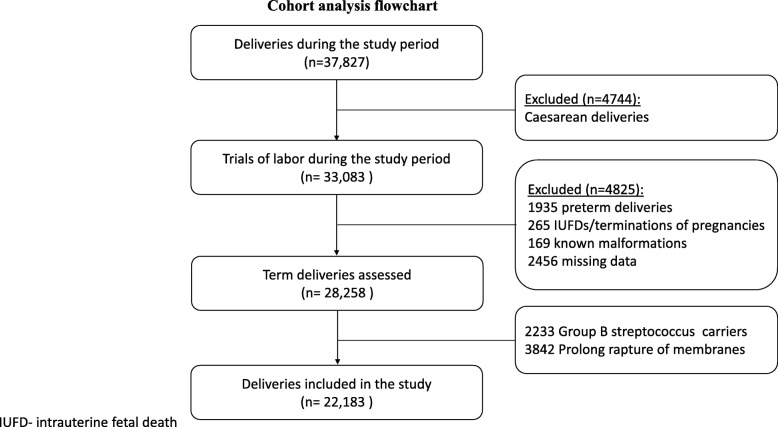
Cohort analysis flowchart

During the study period, a total of 37,827 women delivered at our medical center. Of them, a total of 22,183 women were included in the study (Fig. 1). The cohort was divided according to study groups, as detailed above: up to 4 VEs (*n* = 9716), 5–6 VEs (*n* = 4624), 7–8 VEs (2999), and 9 or more VEs (*n* = 4844). Maternal demographics of the entire cohort are presented in Table 1. The groups differed in duration of labor (up to 4 VEs: 184 ± 122, 5–6 VEs: 349 ± 163, 7–8 VEs: 490 ± 204, 9 or more VEs: 682 ± 308 min, *p* < 0.001) and the time form rupture of membranes to delivery (up to 4 VEs: 180 ± 179, 5–6 VEs: 250 ± 220, 7–8 VEs: 321 ± 231, 9 or more VEs: 422 ± 269 min, p < 0.001).

**Table 1:** Maternal demographics of the study cohort

Demographic data	Up to 4 VEs (*n* = 9716)	5–6 VEs (*n* = 4624)	7–8 VEs (*n* = 2999)	9 or more VEs (n = 4844)	*p*-value
Maternal age (years)	30.1 (5.2)	29.6 (5.5)	29.2 (5.4)	28.7 (5.4)	**< 0.001**
BMI (kg/m^2^)	23.1 (3.6)	23.3 (3.9)	23.3 (3.9)	23.5 (4.1)	**< 0.001**
Obesity	508 (5.5)	251 (5.4)	180 (6.0)	369 (7.6)	**< 0.001**
Gestational age (weeks)	39.6 (1.1)	39.6 (1.1)	39.6 (1.1)	39.6 (1.2)	**< 0.001**
Nulliparity	2306 (23.7)	1727 (31.6)	1563 (52.1)	3521 (72.6)	**< 0.001**
Birthweight (grams)	3271 (407)	3287 (418)	3313 (422)	3333 (599)	**< 0.001**
Small for gestational age	389 (4.1)	173 (3.7)	100 (3.3)	165 (3.4)	0.311
Macrosomia	465 (4.9)	205 (4.4)	169 (5.6)	292 (6.0)	**< 0.001**
Smoking	1046 (10.8)	543 (11.7)	386 (12.9)	1052 (21.7)	**< 0.001**
TOLAC	536 (5.5)	227 (4.9)	179 (5.9)	284 (5.9)	**< 0.001**
Induction of labor	2733 (28.1)	1448 (31.3)	999 (33.3)	2117 (43.7)	**< 0.001**
Epidural	6993 (72.0)	3813 (82.5)	2603 (87.8)	4354 (89.9)	**< 0.001**
Spontaneous ROM	1395 (14.4)	560 (12.1)	348 (11.6)	503 (10.4)	**< 0.001**
Amniotomy	8321 (85.6)	4064 (87.9)	2651 (88.4)	4341 (89.6)	**< 0.001**
Normal vaginal delivery	9377 (96.1)	4320 (93.4)	2729 (91.0)	4105 (84.7)	**< 0.001**
Instrumental delivery	211 (3.9)	304 (6.6)	270 (9.0)	739 (15.3)	**< 0.001**
Diabetes mellitus	625 (6.4)	323 (7.0)	237 (7.9)	406 (8.4)	**< 0.001**
Gestational hypertensive disorders	575 (5.9)	250 (5.4)	185 (6.2)	314 (6.5)	<0.001
Chronic hypertension	45 (0.4)	16 (0.3)	23 (0.8)	32 (0.4)	**0.012**
Labor duration (min)	184 (122)	349 (163)	490 (204)	682 (308)	**< 0.001**
ROM to delivery duration	180 (179)	250 (220)	321 (231)	422 (269)	**< 0.001**

Data are presented as mean (SD) or n (%)

*BMI* body mass index, *TOLAC* Trial of labor after cesarean delivery ROM rupture of membrane, Gestational hypertensive disorders include eclampsia, preeclampsia; and gestational hypertension

Values in bold are statistically significant

A univariate analysis of febrile morbidity complications according to the number of VEs during labor is presented in Table 2. The rate of febrile complications increased with an increase in the number of VEs performed during delivery: intrapartum fever (up to 4 VEs: 0.23%, 5–6 VEs: 0.77%, 7–8 VEs: 2.16%, 9 or more VEs: 4.54%, *p* < 0.001), chorioamnionitis (up to 4 VEs: 0.87%, 5–6: 1.55%, 7–8 VEs: 2.63%, 9 or more VEs: 7.03%, *p* < 0.001), postpartum fever (up to 4 VEs: 0.57%, 5–6 VEs: 1.23%, 7–8 VEs: 2.23%, 9 or more VEs: 2.86%, *p* < 0.001), and endometritis (up to 4 VEs: 0.07%, 5–6 VEs: 0.11%, 7–8 VEs: 0.17%, 9 or more VEs: 0.29%, *p* < 0.001). The rates of intrapartum febrile morbidity (up to 4 VEs: 1.11%, 5–6 VEs: 2.32%, 7–8 VEs: 4.8%, 9 or more VEs: 11.58%, *p* < 0.001), postpartum febrile morbidity (up to 4 VEs: 0.65%, 5–6 VEs: 1.37%, 7–8 VEs: 2.45%, 9 or more VEs: 3.18%, p < 0.001), and peripartum febrile morbidity (up to 4 VEs: 1.76%, 5–6 VEs: 3.67%, 7–8 VEs: 7.2%, 9 or more VEs: 14.73%, p < 0.001) were also differed between the groups.

**Table 2:** Univariate analysis of febrile morbidity complications according to the number of vaginal examinations during labor

Complications	Up to 4 VEs (*n* = 9716)	5–6 VEs (*n* = 4624)	7–8 VEs (*n* = 2999)	9 or more VEs (*n* = 4844)	*p*-value
Intrapartum fever	23 (0.23)	36 (0.77)	65 (2.16)	220 (4.54)	**< 0.001**
Chorioamnionitis	85 (0.87)	72 (1.55)	79 (2.63)	341 (7.03)	**< 0.001**
Intrapartum febrile morbidity	108 (1.11)	108 (2.32)	144 (4.8)	561 (11.58)	**< 0.001**
Postpartum fever	56 (0.57)	57 (1.23)	67 (2.23)	139 (2.86)	**< 0.001**
Endometritis	7 (0.07)	5 (0.11)	5 (0.17)	14 (0.29)	**< 0.001**
Postpartum febrile morbidity	63 (0.65)	62 (1.37)	72 (2.45)	153 (3.18)	**< 0.001**
Any febrile morbidity	171 (1.76)	170 (3.67)	216 (7.2)	714 (14.73)	**< 0.001**

Data are presented as n (%)

Values in bold are statistically significant

Logistic regression models in which febrile morbidity complications served as the dependent variables are presented in Table 3. We found that performing 5 or more VEs during labor was independently associated with intrapartum febrile morbidity (5–6 VEs: aOR = 1.83, 95% CI (1.29–2.61), 7–8: aOR = 2.65 95% CI (1.87–3.76), 9 or more VEs: aOR = 3.47 95% CI (2.44–4.92)), postpartum febrile morbidity (5–6 VEs: aOR = 1.29, 95% CI (1.09–1.86), 7–8 VEs: aOR = 1.94 95% CI (1.33–2.83), 9 or more VEs: aOR = 1.91 95% CI (1.28–2.82)), and peripartum morbidity (5–6 VEs: aOR = 1.48, 95% CI (1.15–1.91), 7–8 VEs: aOR = 2.15 95% CI (1.66–2.78), 9 or more VEs: aOR = 2.57 95% CI (1.97–3.34)).

**Table 3:** Odd ratios for febrile morbidity complications in a logistic regression model

Number of VEs	Intrapartum febrile morbidity		Postpartum febrile morbidity		Peripartum febrile morbidity	
	Unadjusted OR	Adjusted OR	Unadjusted OR	Adjusted OR	Unadjusted OR	Adjusted OR
**Up to 4**	1	1	1	1	1	1
**5–6**	**2.85 (2.03–4.02)**	**1.83 (1.29–2.61)**	**1.74 (1.22–2.48)**	**1.29 (1.09–1.86)**	**2.23 (1.74–2.86)**	**1.48 (1.15–1.91)**
**7–8**	**5.81 (4.19–8.03)**	**2.65 (1.87–3.76)**	**3.19 (2.27–4.48)**	**1.94 (1.33–2.83)**	**4.37 (3.45–5.54)**	**2.15 (1.66–2.78)**
**9 or more**	**12.81 (9.67–16.95)**	**3.47 (2.44–4.92)**	**4.09 (3.06–5.48)**	**1.91 (1.28–2.82)**	**8.19 (6.71–10.01)**	**2.57 (1.97–3.34)**

“Up to 4 VEs” serves a reference. Data are represented as OR (95%CI)

OR was adjusted for maternal age, diabetes mellitus, BMI, gestational age, type of delivery, duration of labor, and the time form rupture of membranes to delivery

Values in bold are statistically significant

When VEs was analyzed as a continuous variable, each VE above 4 raised the risk for intrapartum febrile morbidity by 2%, for postpartum febrile morbidity by 1%, and for peripartum febrile morbidity by 1%.

## Discussion

### Main findings

In the current study, we have demonstrated that the performance of five or more VEs during labor was associated with an increased risk for all febrile morbidities (Table 3). We also found that for intrapartum and peripartum febrile morbidity, the risk is greater as the number of VEs increases.

### Strengths and limitations

The current study is notable for several points of strength. First, to the best of the authors’ knowledge and based on literature search, it is the largest cohort to date (almost 24,000 patients) to examine the association between febrile morbidity and VEs during delivery. Second, by reviewing a minimal 48-h postpartum course, we were able to include postpartum febrile morbidity as an outcome. Third, by performing multivariant analysis, we addressed possible confounders, such as the duration of labor, which allows us to study the independent association between the number of VEs and delivery complications.

Our study is not without limitation, as it is mainly limited by its retrospective design, which might pose a potential bias: it is reasonable to assume that women who became febrile in labor may have been monitored more closely and as a result underwent more VEs. That may potentially be interpreted as a cause and effect, even though the predictor and outcome are actually reversed in that hypothetical scenario. Although it may have affected the results of intrapartum febrile morbidity, postpartum febrile morbidities were analyzed after the exclusion of intrapartum febrile patients, hence were not affected.

Another limitation is that we did not analyze the effect of the time interval between VEs to delivery: it is reasonable to assume that a VE performed far from delivery carries a higher risk for ascending infection than a VE performed close to delivery. However, we managed to partially overcome this confounder by including labor duration in the multivariant analysis.

Third, we are aware of the possibility of VEs which were performed but not electronically recorded, thus were not included in the statistical analysis. Yet, since according to our departmental protocol it is mandatory to report every examination (including cases of re-examination by a second medical crew member for validation of the former), it’s safe to assume that missing data are rare.

### Interpretation

Multiple VEs have been proposed as a risk factor for both chorioamnionitis and postpartum endometritis, as they increase the exposure of the uterine cavity to the vaginal flora [6, 7, 13]. Soper et al. [8] prospectively evaluated 5399 patients for risk factors for the development of intraamniotic infection. In addition to prolonged rupture of membrane and the use of internal monitor, the authors reported that performing more than four VEs during labor at term independently triples the risk for intraamniotic infection. Seaward et al. [9] retrospectively determined predictors for the development of chorioamnionitis and postpartum fever in 5028 patients with premature rupture of membranes at term. The authors found that an increased number of VEs is a risk factor for chorioamnionitis. On the other hand, Cahill et al. [12], who retrospectively estimated the association between number of cervical examinations and risk of maternal fever during term labor, failed to demonstrate this correlation, and reported that the number of VEs during labor was not associated with an increased risk of maternal infection (*n* = 2395). Notably, the latter consisted of a relatively small sample size, and included only intrapartum and early postpartum (up to 6 hours after delivery) febrile morbidities. Therefore, it is safe to assume that our results, which are based on a large cohort and a longer postpartum follow-up, validate the potential risk of multiple VEs for febrile morbidity, as was formerly discussed [8, 9].

The study groups differed significantly in potential confounders: prolonged rupture of membranes, obesity, and diabetes mellitus are known risk factors for febrile morbidity; Epidural anesthesia is associated with increased body temperature; Patients who undergo induction of labor are often being examined before reaching the stage of active labor. Induction of labor is therefore associated with more VEs than spontaneous labor. Therefore, the results of the current study should be interpreted with caution. In order to minimize the risk for bias, and to explore the independent association between the number of VEs and the risk for febrile morbidity, a regression analysis model was performed, in which carefully selected potential confounders were adjusted for.

## Conclusions

The number of VEs performed during labor directly correlates with febrile morbidity. Although, in clinical practice it seems difficult to isolate the number of VEs as a stand- alone variable, after careful statistical analysis, it was found that performing 5 VEs or more independently increased the risk for febrile morbidity. For intrapartum and peripartum febrile morbidity the risk was higher with an increase in the number of VEs.

This data should be taken into account when considering the benefits and necessity of multiple VEs, and should be considered by obstetricians while deciding regarding the number of VEs to perform during labor. This data can also assist organizations and institutions in the establishment of local protocols, to guide caregivers regarding the number and frequency of VEs to be performed in labor.

## Data Availability

The datasets generated and/or analysed during the current study are not publicly available due to medical center’s policy, but are available from the corresponding author on reasonable request.

## Abbreviations

VE: Vaginal examination
BMI: Maternal body mass index

## References

[1] Downe S, Gyte GM, Dahlen HG, Singata M. Routine vaginal examinations for assessing progress of labour to improve outcomes for women and babies at term. Cochrane Database Syst Rev. 2013:7 [cited 2018 13 Oct]. 10.1002/14651858.CD010088.pub2 23857468

[2] de Klerk HW, Boere E, van Lunsen RH, Bakker JJH (2018). Women’s experiences with vaginal examinations during labor in the Netherlands. J Psychosom Obstet Gynecol.

[3] (UK) NCC for W and CH. Normal labour: first stage. 2007 [cited 2018 13 Oct].

[4] Borders N, Lawton R, Martin SR (2012). A Clinical Audit of the Number of Vaginal Examinations in Labor: A NOVEL Idea. J Midwifery Womens Health.

[5] Newton ER. Chorioamnionitis and intraamniotic infection. Clin Obstet Gynecol, 1993. 36(4):795–808 [cited 2018 1 Nov]. 10.1097/00003081-199312000-00004 8293582

[6] Gibbs RS, Duff P (1991). Progress in pathogenesis and management of clinical intraamniotic infection. Am J Obstet Gynecol.

[7] Duff P. Maternal and perinatal infection — bacterial [internet]. Seventh Ed. Obstetrics: Normal and Problem Pregnancies. Elsevier Inc.; 2012. 1140–1155 p.

[8] Soper DE, Mayhall CG, Froggatt JW, Baker ME, Hopwood H, Nesbitt T (1996). Characterization and control of intraamniotic infection in an urban teaching hospital. Am J Obstet Gynecol.

[9] Seaward PG, Hannah ME, Myhr TL, Farine D, Ohlsson A, Wang EE (1997). International multicentre term prelabor rupture of membranes study: evaluation of predictors of clinical chorioamnionitis and postpartum fever in patients with prelabor rupture of membranes at term. Am J Obstet Gynecol.

[10] WHO recommendations: Intrapartum care for a positive childbirth experience. 2018 [cited 2018 13 Oct]. 30070803

[11] Shepherd A, Cheyne H (2013). The frequency and reasons for vaginal examinations in labour. Women Birth.

[12] Cahill AG, Duffy CR, Odibo AO. Number of Cervical Examinations and Risk of Intrapartum Maternal Fever. 2012;119(6):1096–101. 10.1097/AOG.0b013e318256ce3f 22617572

[13] Soper DE, Glen Mayhall C, Dalton HP (1989). Risk factors for intraamniotic infection: A prospective epidemiologic study. Am J Obstet Gynecol.

